# The Prescription of Mobile Apps by Primary Care Teams: A Pilot Project in Catalonia

**DOI:** 10.2196/10701

**Published:** 2018-06-21

**Authors:** Francesc Lopez Segui, Carme Pratdepadua Bufill, Nuria Abdon Gimenez, Jordi Martinez Roldan, Francesc Garcia Cuyas

**Affiliations:** ^1^ TIC Salut Social Generalitat de Catalunya Mataró, Barcelona Spain; ^2^ Centre for Research in Health and Economics Department of Experimental and Health Sciences Universitat Pompeu Fabra Barcelona Spain; ^3^ Universitat de Vic – Universitat Central de Catalunya Vic Spain

**Keywords:** mobile apps, apps, mHealth, primary health care, telemedicine, telemonitoring

## Abstract

**Background:**

In Catalonia, the Fundació TIC Salut Social’s mHealth Office created the AppSalut Site to showcase to mobile apps in the field of health and social services. Its primary objective was to encourage the public to look after their health. The catalogue allows primary health care doctors to prescribe certified, connected apps, which guarantees a safe and reliable environment for their use. The generated data can be consulted by health care professionals and included in the patient's clinical history. This document presents the intervention and the major findings following a five-month pilot project conducted in the Barcelona area.

**Objective:**

The objective of the pilot study was to test, in a real, controlled environment, the implementation of AppSalut. Specifically, we tested whether (1) the procedures corresponding to the prescription, transmission, and evaluation of the data functions correctly, (2) users interact successfully and accept the tool, and (3) the data travels through existing pathways in accordance with international standards. The evaluation is not based on clinical criteria, but rather on the usability and technological reliability of the intervention and its implementation in the context of primary care.

**Methods:**

The project was presented to the Primary Care Team participants to encourage the involvement of doctors. The study involved at least 5 doctors and 5 patients per professional, chosen at their discretion and in accordance with their own clinical criteria. An initial consultation took place, during which the doctor discussed the pilot project with the patient and recommended the app. The patient was sent a text message (SMS, short message service) containing an access code. When the patient arrived home, they accessed their personal health record (PHR) to view the recommendation, download the app, and enter the access code. The patient was then able to start using the app. The data was collected in a standardized manner and automatically sent to the system. In a second visit, the patient looked at the data with their doctor on their clinical station screen. The latter was able to consult the information generated by the patient and select what to include in their electronic health record. In order to assess the performance of the system, three focus groups were performed and two ad-hoc case-specific questionnaires, one for doctors and one for patients, were sent by email. Response was voluntary.

**Results:**

A total of 32 doctors made 79 recommendations of apps to patients. On average, the patients uploaded data 13 times per prescribed app, accounting for a total of 16 different variables. Results show that data traveled through the established channels in an adequate manner and in accordance with international standards. This includes the prescription of an app by a doctor, the patient accessing the recommendation via the PHR, app download by the patient from the official app stores, linking of the patient to the public platform through the app, the generation and visualization of the data on the primary care workstation, and its subsequent validation by the clinician.

**Conclusions:**

First, the choice of apps to be used is fundamental; the user's perception of the utility of the proposed tool being paramount. Second, thorough face-to-face support is vital for a smooth transition towards a more intense model of telemedicine. Last, a powerful limiting factor is the lack of control over people’s ability to use the apps.

## Introduction­­

### Background

As part of the Catalan Ministry of Health, the *Fundació TIC Salut Social* (the ICT Social Health Foundation) works to promote the use of information and communication technologies (ICT) in the field of public health and social welfare. Its main tasks include the observation of new trends, monitoring of emerging initiatives and the provision of services to certify products, systems and apps. In this context, the mHealth Office was created in early 2016 with the aim to bring patients closer to health and social mobility services so that they can interact with the health system in a trouble-free, personal way. The Office created a website featuring mobile apps in the field of health and social care for medical professionals and members of the public, a mechanism for accrediting apps, the development of mobility standards and a means to monitor experiences with health-related apps. As part of the framework of services that meet the corresponding interoperability standards, the Digital Health Platform (DHP) acts as a repository for this information. It also facilitates interactions between members of the public (providing the user with the information they have generated through the use of one or more recommended apps) and doctors (providing support in monitoring the patient’s status and allowing the treatment to be p­ersonalized and adaptable to their needs).

### App Catalogues - The AppSalut Site

Easy to get, easy to use and insanely cheap. The use of mobile apps for health management has been promoted both by independent reviews and public initiatives [1]. In the former, the app stores themselves (Google, Apple, Windows, Amazon, Blackberry) rank the apps in their catalogs according to the opinions of expert or user ratings [2] [3]. Nevertheless, they are unable to avoid significant heterogeneity in their quality [4] and safety standards [5].

Likewise, countless leading websites feature health apps, either exclusively, such as iMedicalApps and *Fundación iSYS’s* iSYScore [6], or only as one of their numerous areas of interest (Android Authority, ForbesTech). As for catalogues aimed at the public sector, we find fewer initiatives: for example, the United Kingdom promotes its “Digital Apps Library,” which is still at the design stage; at the regional level, the Andalusian Health Service maintains its “Catalogue of mobile health apps” recipients of its *AppSaludable* label, its own accreditation process.

In Catalonia, the *Fundació TIC Salut Social’* s mHealth Office created the AppSalut Site as a central, wide-reaching project. It is intended as a showcase of mobile apps in the field of health and social services with its primary objective being to encourage members of the public to look after their health. All of the apps on the website, which are available for both iOS and Android devices for free in leading app stores, need to have passed the Foundation’s own quality control process, which guarantees a safe and reliable environment for their use. In addition, doctors in Primary Care Teams can recommend the apps to their patients in their surgery, thus complementing the follow-up of the patients’ condition by monitoring the data generated, which can be consulted by health care professionals and included in their clinical history, with the patient’s permission. In the future, the plan is to extend access to professionals from other specialties.

After conducting various tests in the preproduction environment, the need arose to carry out a controlled pilot study with doctors and real patients to ensure the system worked correctly from a technological point of view. This document is intended to present its significant findings, generating pioneering evidence for the integration of mHealth technologies with Primary Care systems in a public setting [7,8].

### Objectives of the Pilot Project

**Figure 1:**
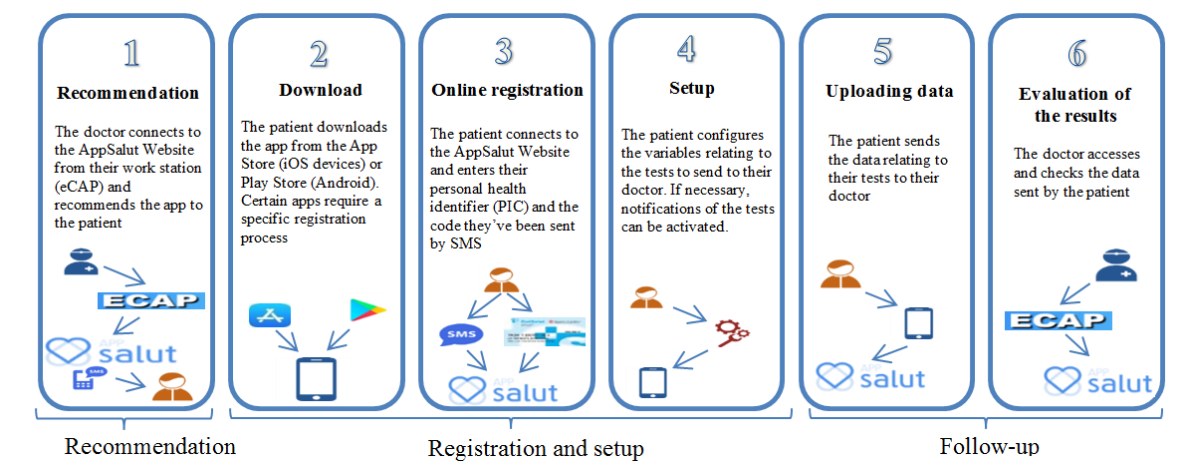
The AppSalut Site recommendation process.

**Figure 2:**
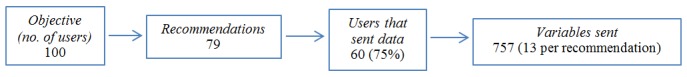
Participant attrition diagram.

The overall objective of the pilot project was to test, in a real, controlled environment, the implementation of AppSalut. Specifically, the three objectives that were to be evaluated are the following: (1) that procedures corresponding to the prescription, transmission and evaluation of the data functions correctly (Figure 1), (2) users interact successfully and accept the tool, and (3) the data travels through existing pathways in accordance with international standards.

The evaluation of the pilot project is not based on clinical criteria but rather the usability and technological reliability of the intervention and its implementation in the context of primary care.

## Methods

### Preliminary Design

The project was presented to the Primary Care Team (PCT) participants to encourage the involvement of doctors. Once they were recruited, together with their corresponding patients, the following three steps were taken. First, an initial consultation took place, in which the doctor was able to explain to the patient what the pilot project consisted of, provide them with the necessary documentation, and recommend the app to them. The patient was sent a SMS text message (short message service, SMS) containing an access code, and the recommendation of the app was registered on the system. Second, when the patient arrived home, they accessed the *La Meva Salut* (My Health, MH) Personal Health Record to view the recommendation, download the app and enter the activation code they were sent by SMS to link the app to the platform. The patient was then able to start using the app. The data was collected in a standardized manner and automatically sent to the system. Lastly, in a second visit, the patient looked at the data with their doctor on their screen. The latter was able to consult the information generated by the patient and select what to include in their Electronic Health Record.

### Participant Recruitment

The agents involved in the execution of the pilot project are presented in Table 1.

The following four PCTs participated in the study and belong to the South Metropolitan Territorial Management (province of Barcelona): Vinyets PCT (Sant Boi de Llobregat), Sant Andreu de la Barca PCT, Cubelles-Cunit PCT and Sant Ildefons PCT (Cornellà de Llobregat). For each team, the involvement of at least 5 PCT doctors and 5 patients per professional was requested and chosen at their discretion and according to their own clinical criteria. Thus, the objective recruitment population was 20 doctors and 100 patients. Patients were selected according to the following inclusion criteria: the patient's expressed their willingness to participate, and proof that they were over the age of consent and gave written authorisation. Since the object of the study was the implementation of the process rather than the evaluation of any clinical outcomes, the intervention was not assigned in a randomized manner. Consequently, potential biases (medium-advanced users of mobile technology) can be assumed to be present and caution should be exercised in the extrapolation of the results.

### Mobile Apps

Three mobile apps were used (see Table 2). They were chosen for their potential in providing continuity of care for the conditions they address, and their ability to adapt to the requirements that were identified during the pilot study. The integrated apps were required to perform specific technical adaptations to conform to the specific doctor-patient context effectively. The three of them could be downloaded free-of-charge.

### Duration

The planned duration was initially 3 months. However, due to the decrease in the use of the platform during the summer holidays (July and August) it was extended to 5 months from the time the PCT candidates for participation in the pilot were identified, until the last recommendation was made. The variables sent by the app continued to be recorded in November to include information from users recruited during the end of October. Thus, the period covers June 1 to November 30, 2017.

### Follow-Up and Monitoring

To initiate the pilot study, presentations were organized to introduce the doctors to the project and to train them in the prescription and use of the apps. Once started, the timing of periodic follow ups was established, accompanied by training for the doctors to ensure the processes were clear and to deal with any doubts. After detecting problems in various phases of the recommendation of the apps, it was decided to occasionally assist the professionals in the prescription process and the patients in downloading, registering, and configuring the app.

**Table 1:** The roles of organizations participating in the study.

Organization	Role
*Fundació TIC Salut Social* (ICT Social Health Foundation)	Coordinated the entire pilot phase, training the doctors, monitoring the project, dealing with any incidents which arose, and the utilization and evaluation of the results
Four Primary Care Teams belonging to the South Metropolitan Territorial Management Directorate of the Catalan Health Institute	Clinicians are the users of the eCAP, the main clinical management software used by primary care clinicians in the public system
The *Dirección Asistencial* ICS (ICS Health Care Directorate)	Participated in the validation of the process and provided training to the professionals involved
The Catalan Personal Health Record *La Meva Salut* My Health (MH)	The functional and technical managers established the AppSalut Site service within MH and provided access to the participating providers in the pilot study
IN2, an app and web developer	Offered support for technological incidents

**Table 2:** Apps used in the pilot project.

App	Health indication	App description
AsmaProcare	Asthma	An app which serves as an instrument for sending information from asthma sufferers to their physician. The daily readings of *PeakFlow* measurements are introduced via the app, as well the use of any rescue medication; the data can be displayed visually in the apps’ interface. In addition, the user is able to see the ongoing treatment introduced in the *backoffice* by their doctor.
ExpertSalud	Chronicity	An app that allows the monitoring of the pharmacological adherence to a treatment. It is designed to help manage the intake of medications, the setting of reminders and the monitoring of variables such as weight and glucose levels.
Sideal	Alcohol consumption	A self-help system for people with alcohol dependence that offers advice aimed both at reducing intake and abstinence and which allows monitoring both of consumption and therapeutic compliance based on the objectives that the person sets or agrees with their doctor.

The recommendation was made to the patient in the doctor’s surgery, while the process of downloading the app, registering the patient on the platform and configuring the relevant variables was carried out in an adjoining surgery by the Foundation's staff. In this way, the most critical period within the process was reduced, where the highest rate of loss of patients was identified: when the user connects with the platform and needs to configure the variables. These follow-ups were an important aspect in the development of the project since they allowed to gain personal experience from the problems which arose and to implement corrective measures and additional training. The following Figure 2 summarizes the evolution of the project’s adherence.

## Results

### Usage of the Instrument

A total of 32 doctors made 79 recommendations of apps to patients, representing 160% of doctors and 79% of recommendations compared to what was expected during the design of the pilot project. Of the recommendations, 75% (60/79) of patients used the app, sending a total of 757 variables to the system (Table 3). In general, the main reasons for non-use were connection errors with the platform, problems with accessing the app (incorrect configuration, failing to activate alerts and problems with the confirmation email), and dropping out (mainly due to the study coinciding with the summer holidays and loss of motivation).

**Figure 3:**
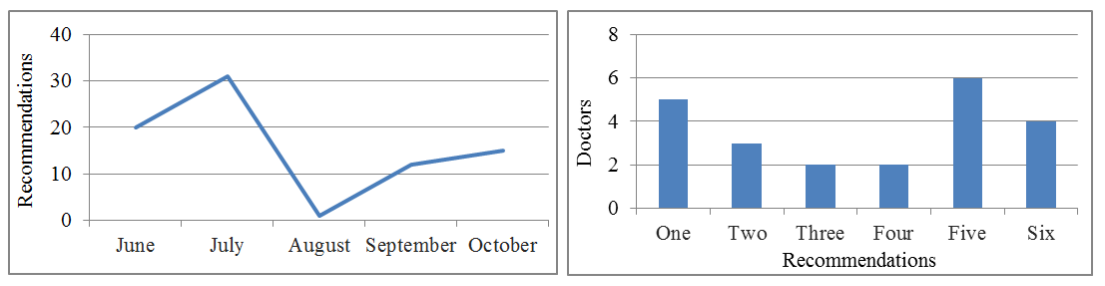
Monthly recommendations and number of recommendations per professional.

The amount of prescriptions made to patients was heterogeneous along the intervention period. As shown by Figure 3, the reduction occurred from the second half of July to the first half of September, coinciding with the doctors’ vacation period, bringing out one of the major design drawbacks. The aforementioned heterogeneity can also be noted by the number of recommendations made by the doctors. A first subgroup has made only one, while at the other extreme many doctors made five or more. It can be seen, therefore, that the shortfall in meeting the objective concerning patient recruitment is primarily due to the first subgroup of less-motivated doctors.

**Table 3:** Physician recommendations, messages, and data-uploads sent per primary care team.

Primary care team	Physician recommendations per primary care team, n	Patients who sent data per primary care team, n (%)	Total messages sent per primary care team, n	Messages per patient per primary care team, n (%)
Cubelles-Cunit	12	10 (83)	206	21 (10.2)
Cornellà de Llobregat	16	12 (75)	165	14 (8.5)
Sant Andreu de la Barca	22	14 (64)	92	7 (7.6)
Sant Boi de Llobregat	29	24 (83)	294	12 (4.1)
Total	79	60 (75)	757	54 (7.1)

**Table 4:** Apps use by patients.

App and variable upload	Number of patients used (n=79), n (%)
AsmaProcare	9 (11)
ExpertSalud	67 (85)
Sideal	3 (4)

### Uploading of Data

Regarding which apps were likely to be prescribed (Table 4), ExpertSalud was the most-used by the patients (67/79, 85%). According to the results of the focus groups and the periodic follow-ups, this could be due to the full range of variables that the app can handle (glycemia, blood pressure, weight, pain, stress, pulse, dizziness). Very few patients used the AsmaProcare app (9/79, 11%), while prescription of the Sideal app was negligible (3/79, 4%).

On average, the patients uploaded data 9.6 (757/79) times per prescribed app, accounting for a total of 16 different variables. Weight was the most used (28/79, 35%) upload, followed by systolic and diastolic blood pressure variables (21/79, 26%) uploads, and heart rate (11/79, 13%) uploads. Other variables such as temperature, alcohol intake and dizziness levels were not used as much, with migraine being the least used variable. To ensure a consistent exchange of the apps’ variables, the mHealth Office and the Foundation's *Oficina de Estándares e Interoperabilidad* (Standards and Interoperability Office) employed a subset of codes for mHealth variables using controlled vocabulary that guarantee their unique identification: Systematized Nomenclature of Medicine-Clinical Terms (SNOMED CT) is the reference vocabulary used in the subset, but other classifications and terminologies, such as International Statistical Classification of Diseases, Ninth Revision, Clinical Modification (ICD-9-CM), International Statistical Classification of Diseases, Tenth Revision (ICD-10), Logical Observation Identifiers Names and Codes (LOINC), *Anatomical*, *Therapeutic*, *Chemical* Classification System (ATC), and International Classification of Primary Care (ICPC), were also considered and then transformed into SNOMED CT.

### Usability Evaluation

Two ad-hoc case-specific questionnaires, one for doctors and one for patients who had participated in the pilot study were sent by email during the first week of November (the deadline for making new recommendations, but while data was still being sent). They were completed on a voluntary basis. A total of 17/32 (53%) responses from doctors and 30/79 (38%) from patients were received.

#### Doctors’ Questionnaire

Information was collected from the doctors as to the app recommendation process, the use of the data, and the general characteristics of the platform. The main ideas derived from each of these issues are described in the following. First, making a recommendation is not an easy task (since it lengthens the consultation) and the patient is not autonomous in its management: they must receive support. Second, visualizing the data is easy and is carried out before, during and after consultation with the patient, indicating the success of the integration model in patient-doctor environments.

**Table 5:** Incidences by type.

Source		Incidence (N=34), n (%)
**AsmaProcare (app)**		
	User training	1 (2.9)
**ExpertSalud (app)**		
	Sign up email	6 (17.6)
	Make of smartphone	2 (5.9)
**AppSalut (site)**		
	SMS^a^ code	16 (47.1)
	Error connecting with platform	3 (8.8)
	Problems connecting patients	6 (17.6)

^a^SMS: short message service.

Third, with respect to the platform, the doctors would like to be able to create alerts based on the data they have received. In general, they value the usefulness of the AppSalut Site as part of the health care process (3.4, on average, on a scale of 1 to 5, 5 being “Very useful”).

#### Patients’ Questionnaires and Focus Groups

Regarding the users, the questionnaire collected information on three topics: the recommendation process, the use of the Personal Health Record, and the use of the app. In relation to the first, the patients’ experience confirms the difficulties identified in linking the app with the professional’s prescription. Nevertheless, the added value that is expected both from the patient’s ability to use the app autonomously and from the doctor’s ability to access the information is very high. With respect to My Health (MH), in spite of the difficulties patients faced in terms of access, the prescription of the app was closely associated with access to the patient’s Personal Health Record (two out of three patients used it). Finally, regarding the usage of the app, patients would consider recommending it to other patients with the same medical condition and generally see it as very simple to use. In general, they rate the AppSalut Site as “very interesting” (4.5, on average, on a scale of 1 to 5).

To complement this qualitative vision, three focus groups were established (two consisting of doctors, one of patients) with a minimum of three participants, with a professional from the Foundation acting as moderator with a script and prepared questions. Both the script and the full summary of the three sessions can be found in the Multimedia Appendix 1 and Multimedia Appendix 2, confirming the ideas collected by the questionnaire’s approach.

### Incidents Detected During the Use of the Platform

During the pilot project, 34 incidents were registered (see Table 5). Two types stand out. First, those related to the AppSalut Site, such as the SMS being sent with a missing or invalid activation code or incidents related to connection and upload errors. Second, those related to the user’s connection with the app (for example, not receiving the confirmation email to be able to use the app).

Concerning the duration of the pilot study, initially, problems were detected related to users getting lost on the platform and their access to the website, requiring a two-day halt in the prescription process. In general, incidents were detected regarding the connection and access to the AppSalut Site that will need to be reviewed during an additional technical audit.

## Discussion

### Principal Findings

In terms of the specific objectives, the execution of the pilot study has shown that, despite the aforementioned incidents, the platform operates continuously and safely: Therefore, it represents a significant experience in the prescription of health apps and the integration of its information in primary care practitioners’ workstation in a public setting. It was observed that it is generally usable although critical issues have been identified in the user experience, which indicates room for improvement. The pilot study showed that the data travels through the established channels in an adequate manner and in accordance with international standards.

The results validate, in a controlled environment with real participants, the entire process. This includes the prescription of an app by a doctor, the patient accessing the recommendation via their Personal Health Record, download of the app by the patient from the official app stores, linking of the patient to the public platform through the app, the generation, visualization of the data on the primary care workstation, and its subsequent validation by the clinician. The following are the major findings. First, shortcomings in terms of the usability of the tool are largely associated with the user linking to the platform using the code sent by SMS, which the patient needs to activate the app. In many instances, the user registration within some of the apps is not intuitive, an issue which is also found when the patient registers with the AppSalut Site using the SMS code: some users are unable to follow the process. Once this barrier is overcome, the process is smooth in terms of sending and viewing data. Second, the patients have a very high opinion of the service while the doctors feel they need to carefully manage its implementation so as not to overload themselves: for the former it is an additional service, for the latter an added burden. Related to this, the registration process within the app and signing up with the platform is perceived as much more critical for the doctors than for the patients, who are less concerned about the difficulties. For both groups, the integration of the data generated in their usual interfaces is a key factor in its acceptance. Third, there is a steep learning curve associated with the entire process of the use of such mobile technologies; in many cases the doctors ask for additional training for the apps, both for themselves and for the patients, potentially people aged over 65, where the digital divide is present. Fourth, the recurring need for reviews and support for doctors and patients indicates that support elements are needed at least in the early stages of the intervention. Finally, in relation to the information sent by the patients, the doctors feel that it would be more useful if alerts were received within the professional work interface.

### Conclusion

By way of a recommendation, three factors can be identified which would improve similar experiences. First, the choice of apps to be used is fundamental; the user's perception of the utility of the proposed tool being paramount. Second, thorough face-to-face support is vital to a smooth transition towards a more intense model of telemedicine. Last, a powerful limiting factor is the lack of control over people’s ability to use the apps.

## Appendix

Multimedia Appendix 1 Guide to the focus groups.

Multimedia Appendix 2 Summary of focus groups.

## Abbreviations

ATC: Anatomical, Therapeutic, Chemical Classification System
DHP: Digital Health Platform
ICT: information and communication technologies
ICPC: International Classification of Primary Care
LOINC: Logical Observation Identifiers Names and Codes
MH: My Health (Catalan Personal Health Record)
PCT: Primary Care Teams
SMS: short message service
SNOMED CT: Systematized Nomenclature of Medicine–Clinical Terms CIM
