# Supplementary material for: The Prescription of Mobile Apps by Primary Care Teams: A Pilot Project in Catalonia
Source: JMIR Mhealth Uhealth. 2018 Jun 21;6(6):e10701. doi: 10.2196/10701 (PMC6035343; doi:10.2196/10701)
Supplement: Multimedia Appendix 1 [file mhealth_v6i6e10701_app1.pdf]

Multimedia appendix 1

Doctors

Phase 1: Recommendation and downloading of the app

1. Opinion regarding the project
2. What do you think about recommending apps?
3. Making a recommendation, was the process straightforward?
4. Did you help the patient to download and install the app?
5. Does it take long? How long do you think should be necessary?
6. Should it take place in the doctor’s surgery or should it be done at the reception counter?

Phase 2: Accessing data

1. Have you accessed the data collected by the patient?
2. Was it easy to access the data?
3. Did you schedule it? Did you timetable a visit in your diary?
4. Did you find it useful?
5. Have you checked the data?

Phase 3: Healthcare process

1. Do you think that using apps can improve the healthcare process with your patients?
2. Do you think it improves doctor-patient communication?
3. Do you have any suggestions for improvements?

Patients

Phase 1: Recommendation and downloading of the app

1. Opinion regarding the project
2. Do you think the doctor prescribed the app in the right way?
3. Did you find the process straightforward?
4. Were you able to download the app easily?
5. Have you set up the app? Was it easy to set up? Did you need help? Was it difficult?

Phase 2: Accessing data

1. Did you find the app easy to use?
2. Did you activate any alerts (reminders - warning messages)?
3. How often did you check the data?
4. Did you find it useful?
5. Do you have any suggestions for improvements to the app?

Phase 3: Healthcare process

1. Do you think that using apps can improve the healthcare process with your doctor?
2. Do you think it improves communication with your doctor?
3. Do you have any suggestions for improvement
