# Supplementary material for: The Prescription of Mobile Apps by Primary Care Teams: A Pilot Project in Catalonia
Source: JMIR Mhealth Uhealth. 2018 Jun 21;6(6):e10701. doi: 10.2196/10701 (PMC6035343; doi:10.2196/10701)
Supplement: Multimedia Appendix 2 [file mhealth_v6i6e10701_app2.pdf]

Multimedia appendix 2

AppSalut Pilot Focus Group - Doctors

	Positive aspects	Negative aspects	Possible improvements
**Recommendation and installation of the app**	• Chronic patients aged under 60 if they use their smartphone and see the initiative as positive.<br>• The process of recommending an app to a patient is straightforward.	• The process of linking the app to the patient ought to be easier and automated.<br>• In most cases the doctor has to download the app, since the patient doesn't know how to.<br>• Downloading and installing the app takes too long (30 -45 minutes).<br>• There are no minimum requirements or information regarding the characteristics of the patients' smartphone (amount of free space, model, etc.), which makes the installation process more complex.<br>• Chronic patients are usually older and unsure how to use a smartphone, nor do they wish to be monitored by an app.<br>• Reluctance on the part of doctors to processes integrated with ICT: they do not currently work with e-consultation, email, or recommend MH.<br>• The population of patients and doctors is not yet ready to integrate these ICT processes.	• A link needs to be created with the recommendation of the app for the patient and another to the official app stores to download the app.<br>• Enable the accreditation process in the app: one only need enter the SMS code and needn't validate their email and PIC (Personal Identification Code) to connect with the platform (e.g. ExpertSalud)<br>• Carry out the installation of the app and connection to the platform outside the doctor's surgery (reception counter), since the patient does not have sufficient knowledge and the doctor does not have time during the consultation.<br>• Incorporate information as to the project during public information campaigns such as dialling 061 [the emergency number] where information on how to download and set up the app can be included, in order that patients are better informed.
**Accessing data**	• Accessing the data is simple.<br>• Useful for the doctor and the patient.	• Daily workload does not allow time to review and check the data.	• When the data is checked on the platform it could be added automatically to the patient's clinical history data, without the professional having to enter it manually.
**Face-to-face process**	• Integration of mobility data in the patient's Electronic Health Record to monitor the disease.<br>• Raise awareness among patients, helping them become involved in their treatment.<br>• It represents an improvement in the care process because both the doctor and the patient can monitor the information (highly recommended in chronic cases).	• It is a useful tool, for but the process shouldn't solely take place the doctor's visit.<br>• Adds to the doctors' workload rather than being an integrated healthcare process.	• Increase in the number of available apps.<br>• Generate alerts on the eCAP when a patient reports data that exceeds the established parameters, in order to monitor the disease.<br>• Train reception staff in how to download the apps in order to help the patient with the set up.<br>• Patients with diseases that experience flare-ups (Crohn's, ulcerative colitis, etc.), or with exacerbation of their disease, are susceptible to the use of apps that help them monitor the disease. Also, patients with COPD in order to provide compliance advice for inhalers.

AppSalut Pilot Focus Group – patients

	Positive aspects	Negative aspects	Possible improvements
**Recommendation and installation of the app**	• Improves the healthcare process performed by the doctor in terms of monitoring the patient's illness.<br>• Straightforward process.	• The download, connection with the platform and configuration process either needs help from a third party, or there needs to be very clear information on how to perform the procedure.	• Use in-app registration instead of the verification process requiring the sending and receipt of an email (e.g. ExpertSalud).
**Usage of the app**	• The use of the app does not require much knowledge if the patient is well acquainted with their smartphone.	• Patients who have difficulty using their smartphone need additional help from family members to send data.	• The ability to edit data that has already been sent for a particular period of time in case incorrect data has been sent, or the option of deleting and resending it.<br>• Include a two-step verification before the data is sent to ensure it is sent correctly.<br>• Improve the calendar, to include national holidays when configuring the alarms for the variables being monitored.<br>• Allow the option to consult data for specific periods within the app.<br>• Include a confirmation message after sending data to the platform.<br>• Include the possibility of changing the time when the results are sent, since data cannot always be sent when the measurement is made, in order to avoid three consecutive uploads corresponding to morning, afternoon and night.<br>• Include the option of postponing the measurement for a certain period of time.<br>• Improve notifications, with a new reminder sent if a measurement is not uploaded. Review the types of notifications.
**Face-to-face process**	• A valuable tool.<br>• Monitoring of the patient's progress has moved from paper to the smartphone.<br>• Allows the illness to be monitored more effectively.<br>• Both the patient and the doctor are involved.<br>• Improves patient-doctor communication in monitoring the disease.		• Send a message when the doctor accesses or validates the data sent by the patient.<br>• The possibility of sending a message to the doctor to indicate a change in the symptoms which could generate an alarm or an appointment.<br>• To be able to link the use of the app with home visits or non-face to face means of communicating with the doctor.
